# Anti-inflammatory potential of goldenberry-derived exosome-like nanoparticles in macrophage polarization

**DOI:** 10.2144/fsoa-2023-0172

**Published:** 2024-05-15

**Authors:** Vanessa Vanessa, Heni Rachmawati, Anggraini Barlian

**Affiliations:** 1 School of Life Sciences & Technology, Institut Teknologi Bandung (ITB), Bandung, West Java, 40132, Indonesia; 2 Research Center for Nanoscience & Nanotechnology, Institut Teknologi Bandung (ITB), Bandung, West Java, 40132, Indonesia

**Keywords:** anti-inflammatory, goldenberry, *Physalis peruviana*, macrophage polarization, plant-derived exosome-like nanoparticles, PDEN

## Abstract

**Objective:** Overpopulated M1 macrophages can trigger chronic inflammation. Plant-derived exosome-like nanoparticles have been reported to show beneficial bioactivities. **Aim:** To isolate PDEN from goldenberry fruits and evaluate its anti-inflammatory potential in macrophage polarization. **Methods:** GDEN were isolated by centrifugation and precipitation methods. LPS-induced RAW 264.7 cells were treated with GDEN before being evaluated with nitric oxide production assay and flow cytometry of CD80 and CD209. **Results:** GDEN averaged 227.7 nm in size and spherical-shaped. GDEN 40 μg/ml decreased NO production in LPS-induced cells. Flow cytometry showed that CD209 (M2 marker) positive cells were up-regulated after being treated with 20 μg/ml GDEN. **Conclusion:** GDEN showed anti-inflammatory potential through the ability to reduce M1 macrophages product and promote M2 polarization.

## Plain language summary

During inflammation, there are immune cells called macrophages which can act in two different ways. They are M1 cells which drive inflammation and M2 cells which resolve inflammation. The imbalance of M1 and M2 cells can be harmful to the body. For example, having too many M1 macrophages will cause chronic inflammation. Nano-sized particles found in plants are known to have some benefits as future drugs. They have good biocompatibility, can be produced in large quantities and show various bioactivities. Goldenberry has been used as an herb and is known to have good effects on human health, like reducing inflammation. In this study, nanoparticles from goldenberry fruits, also known as goldenberry-derived exosome-like nanoparticles (GDEN), were successfully extracted using a chemical, polyethylene glycol (PEG) 6000. GDEN showed no harmful effect on mice macrophage cells (RAW 264.7) and had been successfully taken inside by the cells. GDEN also showed reducing inflammation potential. This was supported by the decrease of M1 cell product and the increase of M2 cell marker. Hence, GDEN are worthy to be studied further for its usage to treat inflammation and other related health problems.

## Abstract (plain language summary)

The imbalance of M1-M2 proportion can cause several disadvantages to human body and PDEN have been reported to show an effect on macrophage polarization.

GDEN were successfully isolated from *Physalis peruviana* fruits by centrifugation method combined with precipitation using PEG6000 12%.

Characterization by PSA and TEM showed that GDEN averaged 227.7 nm in size and spherical-shaped.

GDEN were not cytotoxic to RAW 264.7 cells until concentration of 60 μg/ml, suggesting its advantage of *in vitro* biocompatibility.

GDEN had been successfully internalized by RAW264.7 during 2 h incubation period and internalization was enhanced with longer incubation period.

Treatment with 40 μg/ml GDEN was able to decrease NO production on LPS-induced cells significantly compared with other GDEN concentrations, indicating the ability of GDEN to suppress inflammation.

Tendency to decrease M1 population was observed on LPS-induced cells after being treated with GDEN although it was not statistically significant.

Treatment with 20 μg/ml GDEN gave the highest M2 population on LPS-induced cells, thus supporting GDEN anti-inflammatory potential.

GDEN showed anti-inflammatory potential by decreasing M1 product and M1 population, along with promoting M2 polarization when treated with 20 μg/ml GDEN.

## Graphical abstract

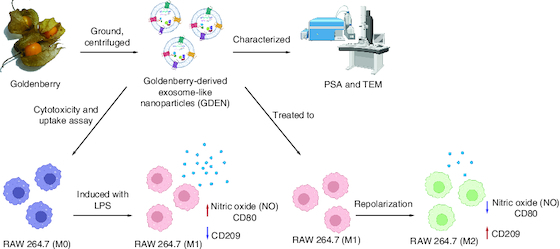

GDEN and Macrophage Polarization (Credit: BioRender).

Inflammation is one of the nonspecific defense mechanisms that intends to decelerate the spread of pathogens, destroy occurring pathogens and facilitate tissue repair, thus restoring homeostasis [1]. During inflammation, the alteration of tissue condition will attract macrophages to the inflammation site. Macrophages act as phagocyte cells that destroy pathogens by lysozyme, produce toxic substances to eliminate targets, and present antigen to T helper cells [1]. As a response to their environment, macrophages can be polarized into two types, M1 macrophages which produce proinflammatory cytokines and M2 macrophages which produce anti-inflammatory cytokines. M1 macrophages are typically stimulated by LPS (lipopolysaccharide), tumor necrosis factor (TNF) and interferon (IFN)-γ, while M2 macrophages are stimulated by IL-4, IL-13 and IL-10 [2,3]. M1 can be identified by its proinflammatory cytokines and chemokines, such as TNF-α, IL-6, IL-12, IFN type I, CXCL1-3, CXCL-5, inducible NO synthase (iNOS) and nitric oxide (NO), along with its surface markers, such as major histocompatibility complex (MHC)-II, CD68, CD80 and CD86 [^2-4^]. Meanwhile, M2 can be distinguished by anti-inflammatory cytokines, such as IL-10, transforming growth factor (TGF)-β, IL-1Ra, CXCR1, CXCR2 and Arg1, along with CD206 dan CD163 [2,3]. Disruption of M1 and M2 proportion can give rise to diseases. For example, overpopulated M1 will trigger chronic inflammation. As a part of regeneration process, long-term exposure to inflammation can hinder the wound healing process by inhibiting re-epithelization and lead to scar formation [4]. Moreover, chronic inflammation itself can increase the prevalence of other diseases which threaten human health [^5-7^].

Exosomes are extracellular vesicles with characteristics and compounds similar to their origin cells, thus they are assumed to be easily recognized and beneficial to recipient cells [8]. Exosomes then are promising to be used as cell-free-based therapy which can reduce the risk of tumor formation and biocompatibility issues [4,9]. Exosomes are frequently isolated from stem cells [3,10]. Its therapeutic potential, for example in dentistry, has been well-reviewed by Ana *et al.* The potential includes effects in regeneration process and it can target various mechanisms in the regeneration phases, such as macrophage polarization, regulation of T-cell activation, angiogenesis etc. [4]. However, the quantity of exosomes that can be isolated from stem cells is limited and the process also requires high cost, as well as ethical problems when using stem cells apart from mesenchymal stem cells [9]. Therefore, the attention to exosomes source shifts to plants. Exosome-like nanovesicles isolated from plants are later known as plant-derived exosome-like nanoparticles (PDEN) [11]. PDEN have similar morphology and composition with mammalian-derived exosomes [8,11]. Compare to mammalian-derived exosomes and direct use of plant extract, PDEN have good biocompatibility, less toxic, low immunogenicity, target specific, able to be produced in large quantities, able to cross blood-brain barrier and good stability in digestive tract [8,12,13]. PDEN are reported to carry lipid, protein, miRNA and chemical substances or metabolites that are specific to their origins [9,12,14]. PDEN have been successfully isolated from some fruits and vegetables, such as grape, lemon, apple, ginger, broccoli, etc. Each of them has been reported to possess beneficial bioactivities to human health [8,12,14]. The effects of PDEN in macrophage polarization have also been shown by ginseng [13,15], turmeric [16] and *Pueraria lobata*-derived nanovesicles [17].

Goldenberry (*Physalis peruviana*), known as “*ciplukan*” in Indonesia, has been consumed as daily food and traditional medicine in some countries, either from its leaves, fruits or stems [18]. Goldenberry has shown several bioactivities which are antiinflammation, antioxidant, anticancer and antimicrobial [^19-23^]. Those bioactivities are likely to be related to its contents, such as vitamin C, B3, carotenoid, phenol, flavonoid, tannin, etc [18,22]. Regarding its anti-inflammatory effect, goldenberry extract obtained from its leaves or fruits has been reported to decrease proinflammatory cytokines and related enzymes in cells [24]. These findings encourage further research about goldenberry therapeutics application, particularly by isolating and examining its PDEN anti-inflammatory potential which is yet to be discovered. Therefore, this research aims to find out whether PDEN can be isolated from goldenberry fruits and have anti-inflammatory potential in macrophage polarization.

## Materials & methods

### RAW 264.7 cells, goldenberry & reagents

Reagents used in this research were phosphate buffer saline (PBS), bicinchoninic acid assay (BCA) kit purchased from Thermo Scientific (MA, USA); Dulbecco's Modified Eagle's Medium (DMEM) – high glucose, dimethyl sulfoxide (DMSO), PKH67, antibiotic-antimycotic (AbAm), paraformaldehyde, lipopolysaccharide (LPS), 3-[4,5-dimethylthiazol-2-yl]-2,5-diphenyl tetrazolium bromide (MTT), dexamethasone (DEX), bovine serum albumin (BSA) purchased from Sigma-Aldrich (MI, USA); fetal bovine serum (FBS) purchased from Gibco (NY, USA), FACS buffer, 4′,6-diamidino-2-phenylindole (DAPI), dan Griess reagent purchased from Invitrogen (MA, USA); FITC anti-human CD80 (305206), dan PE anti-human CD209 (330106) from Biolegend (CA, USA).

RAW 264.7 cells were obtained from School of Life Sciences and Technology, Bandung Institute of Technology (SITH ITB), Animal Cells Culture Laboratory. Cells were cultured in DMEM, FBS 10%, and AbAm 1% and incubated in 37 °C and CO_2_ 5% condition. Medium was changed every 2–3 days and cells were passaged when reaching 70–80% confluency.

Goldenberries (*Physalis peruviana*) were purchased from Bandung plantation online shop. The fruits were stored at 4 °C, intact with its calyx. The fruits in good condition were selected, removed from its calyx, and rinsed before use.

### Isolation & characterization of goldenberry-derived exosome-like nanoparticles

Goldenberry-derived exosome-like nanoparticles (GDEN) isolation protocols were based on Kalarikkal *et al.* with certain modifications. First, goldenberries were removed from its calyx, rinsed and ground with food chopper. The juice was then filtered with sieve, filter paper (2.5 μm) and nylon mesh 100. The juice was centrifuged three-times at 2000× *g* for 10 min, 6000× *g* for 20 min and 10,000× *g* for 40 min in 4 °C condition. Obtained supernatant was mixed with polyethylene glycol (PEG) 6000 12% 1:1 (v/v) and stored in 4 °C chiller overnight. Then, it was centrifuged again at 10,000× *g* for 1 h in 4 °C condition. After centrifugation, supernatant was discarded and pellet was resuspended in double-distilled water, then filtered with syringe filter of 0.22 μm. GDEN were stored at 4 °C until usage. Protein concentration of GDEN was measured with BCA assay. Characterization was done by transmission electron microscope (TEM HT7700) and particle size analyzer (Nano Particle Size Analyzer Horiba SZ-100) to determine its morphology, diameter, polydispersity index (PI) and size distribution.

### Cytotoxicity evaluation of goldenberry-derived exosome-like nanoparticles on RAW 264.7 cells

RAW 264.7 cells were seeded in 96-well plate with density of 10,000 cells/well and incubated overnight to adhere. Cells were treated with 0; 2.5; 5; 7.5; 10; 20; 40; 60; 100 μg/ml GDEN and incubated for 24 h. Then, medium was changed into MTT solution and cells were incubated for 4 h. Formation of crystal formazan was diluted in 100 μl DMSO. Absorbance of solution was measured with microplate reader (Bio-Rad^®^ iMark™ Microplate Absorbance Reader, Japan) at 595 nm.

### Cellular uptake assay of goldenberry-derived exosome-like nanoparticles by RAW 264.7

GDEN were first labeled with PKH67. GDEN (200 μl) were mixed with 1 μl PKH67, and 99 μl Diluen C and incubated for 4 min at room temperature before being centrifugated at 10,000 rpm for 1 h. Supernatant was discarded and pellet was resuspended in DMEM + AbAm 1%. RAW 264.7 cells were seeded in glass bottom dish 35 mm with density of 2 × 10^5^ cells/dish and incubated until adhered to dish. Cells were later treated with 20 μg/ml PKH67-labelled GDEN and incubated for 2 h and 6 h. After incubation, medium was discarded and cells were fixed with 4% paraformaldehyde for 10 min. Cells were washed with PBS three-times and stained with DAPI for 15 min. Cells were washed again with PBS three times, then examined with confocal microscope (Olympus FV-1200, Japan, ITB-Olympus Bioimaging Center).

### Cytotoxicity evaluation of lipopolysaccharide on RAW 264.7 & nitric oxide production assay of LPS-induced RAW 264.7

RAW 264.7 cells were seeded in 96-well plate with density of 10,000 cells/well and incubated overnight. Cells were treated with 0; 0.05; 0.1; 0.2; 0.5; 1; 2.5; 5; 10 μg/ml LPS and incubated for 24 h. Cell viability was assayed with MTT solution. Formation of crystal formazan was diluted in 100 μl DMSO. Absorbance of solution was measured with microplate reader at 595 nm.

In NO production assay, RAW 264.7 cells were seeded in 24-well plate with density of 1.5 × 10^5^ cells/well. Cells were treated with 0, 10, 50, 100, 200 and 500 ng/ml LPS and incubated 24 h. After that, 100 μl medium or supernatant of each well was transferred to 96-well plate. Griess reagent (N-naphthyl-ethylenediamine and sulfanilic acid 1:1) was added to each well with volume of 100 μl and incubated at room temperature for 10 min. Absorbance of solution was measured with microplate reader at 595 nm.

### Nitric oxide production assay of LPS-induced RAW 264.7 treated with goldenberry-derived exosome-like nanoparticles

RAW 264.7 were seeded in 24-well plate with density of 7.5 × 10^4^ cells/well and incubated overnight. Cells were induced with 100 ng/ml LPS to generate M1 phenotype. Cells were then treated with 0; 7.5; 10; 20; 40 μg/ml GDEN, and 1 μg/ml dexamethasone as positive control. After 24 h incubation, each well medium was transferred to 96-well plate. Griess reagent was added to each well with volume of 100 μl and incubated at room temperature for 10 min. Absorbance of solution was measured with microplate reader at 595 nm.

### Analysis of goldenberry-derived exosome-like nanoparticles effect in RAW 264.7 polarization

RAW 264.7 cells were seeded in six-well plate with density of 5 × 10^5^ cells/well and induced with 100 ng/ml LPS suspended in DMEM + FBS 10%. Then, cells were treated with 0, 10, 20, 40 μg/ml GDEN or 1 μg/ml dexamethasone. After 24 h incubation, cells were harvested with cell scrapper, blocked with BSA 3% for 1 h at room temperature, and stained with conjugated antibodies (CD80-FITC and CD209-PE) for 30 min at 4 °C. Cells were washed with FACS buffer two-times, resuspended in 500 μl FACS buffer, and analyzed with flow cytometer (BD FACSLyric, USA).

### Statistical analysis

Results were presented in mean ± standard deviation. Each experiment was done once with three technical replicates (cells were grown in three different wells). Statistical differences were calculated by one-way ANOVA-Dunnett test due to the unequal standard deviation of each group that was being statistically compared. Data were visualized using GraphPad Prism 9.0.0.121 and considered statistically significant if p-value <0.05.

## Results

### Isolation & characterization of goldenberry-derived exosome-like nanoparticles

GDEN were successfully isolated by utilizing differential centrifugation method combined with precipitation using 12% PEG6000. Isolated GDEN were characterized by PSA to measure its size and homogeneity and TEM to observe its morphology. The average GDEN size was 227.7 ± 37.5 nm. GDEN with the size of 218.6 and 246.98 nm had the highest frequency, as shown in Table 1. Another parameter used to determine the quality of PDEN or lipid-based nanocarriers is polydispersity. Polydispersity (PI) is the heterogeneity degree of a group of particles. The good PI for lipid-based nanocarriers is considered to be below 0.3 [25]. In this study, the polydispersity of GDEN was measured to be 0.111 and it fulfilled the standard (<0.3), thus GDEN were considered homogeneous and acceptable in therapeutic application.

**Table 1.** Size distribution of goldenberry-derived exosome-like nanoparticles.

Diameter (nm)	Frequency (%)
171.25	2.552
193.48	15.279
218.60	25.279
246.98	25.973
279.04	19.451
315.27	9.548
356.20	1.265

**Figure 1.**
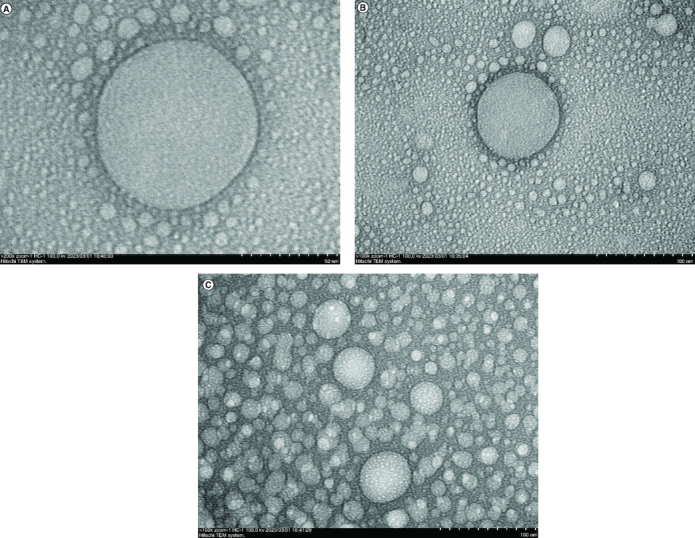
Transmission electron microscopy of GDEN with magnification. 200.000× **(A)**, 100.000× **(B)** and **(C).**

As shown in Figure 1, GDEN was observed to be spherical-shaped and encapsulated by particular membrane. These characteristics are alike to the characteristics of mammalian-derived exosomes [8]. Similar results also have been reported by Ratnadewi *et al.* and Kim *et al.* who isolated PDEN from ginger and ginseng, respectively. The protein concentration of GDEN was measured to be 104.87–190.94 μg/ml. Isolation done in this study yielded 8.89–14.39 mg GDEN per kg goldenberry fruits. This output was lower compared with PDEN isolated from ginseng, ginger and turmeric which yielded 500 mg/kg, 2–4 g/kg and 50–100 mg/kg, respectively [15,16,24]. This could be caused by different methods of isolation used by other authors. Isolation using PEG6000 tended to yield lower PDEN compared with ultracentrifugation [24].

### Cytotoxicity of goldenberry-derived exosome-like nanoparticles on RAW 264.7 cells

**Figure 2.**
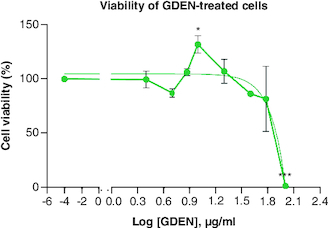
Percentage of RAW 264.7 viability after being treated with GDEN in various concentrations. Each treatment was compared with control, *p-value < 0.05; **p-value < 0.01; ***p-value < 0.001 (n = 3). GDEN: Goldenberry-derived exosome-like nanoparticles.

The viabilities of RAW 264.7 cells treated with various concentrations of GDEN were still above 80%, except concentration of 100 μg/ml, as shown in Figure 2. This result indicated that GDEN were not cytotoxic toward RAW 264.7 cells, at least till concentration of 60 μg/ml. GDEN with concentrations of 7.5, 10 and 20 μg/ml even promoted cell growth, marked by viabilities above 100%. PDEN isolated from other plants, such as turmeric and grapefruit, were not cytotoxic on cells till concentration above 60 μg/ml too [16,26]. Not only *in vitro* but PDEN of turmeric were also not cytotoxic when tested *in vivo* in mice and zebrafish to concentration of 500 μg/ml [16]. These findings support the argument that PDEN are not toxic and have great biocompatibility across species. This advantage is necessary for PDEN's future application as therapeutic medicine.

### Goldenberry-derived exosome-like nanoparticles uptake by RAW 264.7 cells

**Figure 3.**
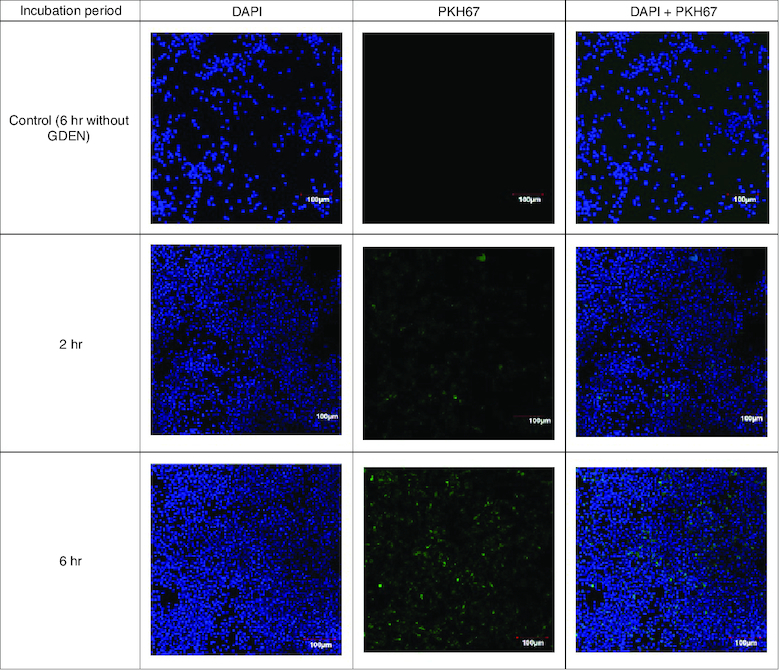
Confocal microscopy of GDEN uptake by RAW 264.7 cells after 2 and 6 h incubation period, magnification of 200×.

**Figure 4.**
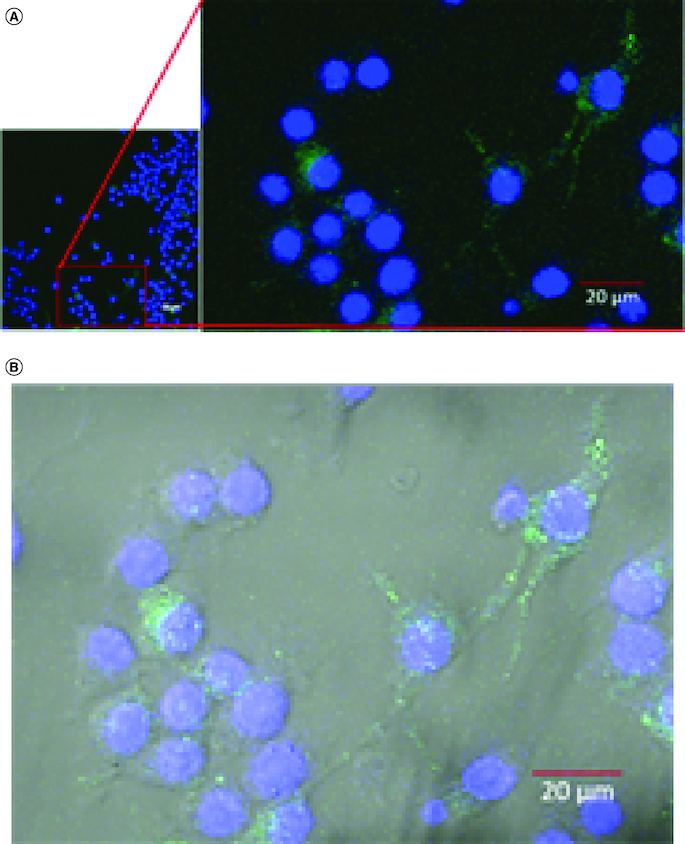
Confocal microscopy of GDEN uptake by RAW 264.7 cells during 6 h incubation period, magnification of 400×. Goldenberry-derived exosome-like nanoparticles (GDEN) (green) was observed around the nucleus (blue) **(A)**, further observation with bright-field overlay confirmed the internalization of GDEN into the cell cytoplasm **(B).**

In cellular uptake assay, cells were incubated with GDEN for 2 h and 6 h at 37 °C. Cells were observed by confocal microscope and results were shown in Figure 3. Green fluorescent from PKH67 had emerged during 2 h incubation period, indicating GDEN had been successfully internalized by RAW 264.7. Internalization of GDEN by cells increased as the incubation period was prolonged. This was observed from the enhancement of PKH67 fluorescent during 6 h incubation period. Internalized GDEN spread throughout cell cytoplasm and around nucleus as presented in Figure 4. The success of GDEN internalization by cells promotes its potential to be used as therapeutics method. The uptake of PDEN by cells is influenced by its lipid and protein composition. The protein will determine its uptake mechanism, while lipid composition determines its uptake efficiency, like membrane fusion, protein binding toward cells and cell target [11,14].

### Cytotoxicity of lipopolysaccharide & nitric oxide production of LPS-induced RAW 264.7

**Figure 5.**
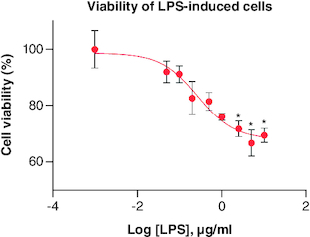
Percentage of RAW 264.7 viability after being treated with LPS in various concentrations. Each treatment was compared with control, *p-value < 0.05; **p-value < 0.01; ***p-value < 0.001 (n = 3). LPS: lipopolysaccharide.

LPS treatment was observed to decrease cell viability along with increasing its concentration, as shown in Figure 5. Cells with viability up to 80% were still found in treatment with 0.05–0.5 μg/ml LPS. Thus, these concentrations were used in the following assay which was NO production assay in order to find out the ability of LPS in inducing inflammation in cells.

**Figure 6.**
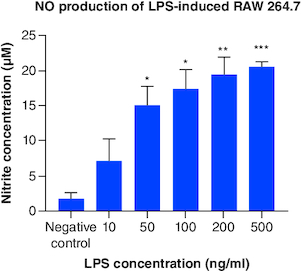
Total NO production from LPS-induced RAW 264.7. Each treatment was compared with control and tested with ANOVA-Dunnett's test, *p-value < 0.05; **p-value < 0.01; ***p-value < 0.001 (n = 3). ANOVA: Analysis of variance; LPS: Lipopolysaccharide; NO: Nitric oxide..

Based on Figure 6, NO production from LPS-induced cells was higher than control and statistically significant starting from concentration of 50 ng/ml. Increasing NO production was also observed along with increasing LPS concentration. This result suggested LPS had successfully induced RAW 264.7 cells to express M1 phenotype.

LPS is the component of Gram-negative bacteria's outer membrane which will be recognized and bind to toll-like receptor 4 (TLR4) on macrophage. Signal generated from TLR4 will produce nuclear factor (NF)-κB which is a factor transcription that regulates the expression of proinflammatory genes in M1 macrophages [2,27]. Besides NF-κB, another factor transcription, signal transducer and activator of transcription (STAT)1, will be expressed too. This factor transcription regulates the expression of iNOS (inducible nitric oxide synthase) [28]. iNOS is often found in M1 as it functions to produce NO from L-arginine and oxygen molecules [29]. During inflammation, NO is produced and considered as radical compound that helps destroy pathogen [1]. Based on this mechanism, further research is recommended to include additional assays, such as iNOS, NF-κB or other proinflammatory cytokines, for example, IL-6 and IL-1β detection, to affirm the success of inducing inflammation.

### Nitric oxide production of LPS-Induced RAW 264.7 after treated with goldenberry-derived exosome-like nanoparticles

**Figure 7.**
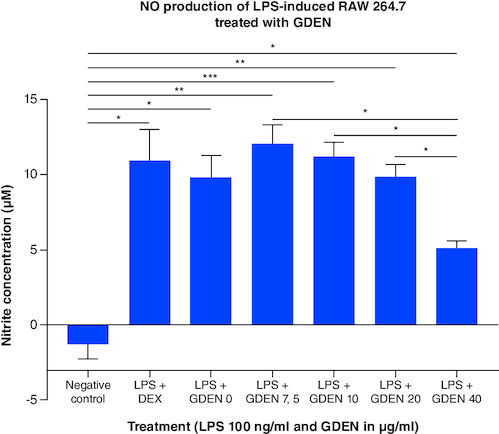
Total NO production from LPS-induced RAW 264.7 after being treated with various concentrations of GDEN. Treatment with 40 μg/ml GDEN decreased NO production significantly compared with other GDEN concentrations. Each treatment was compared with every other treatment and tested with ANOVA-Dunnett's test. *p-value <0.05; **p-value <0.01; ***p-value <0.001 (n = 3).

GDEN were given to LPS-induced cells to examine its anti-inflammatory potential, marked by decreasing the production of NO through promoting M1 polarization into M2. Based on Figure 7, higher concentration of GDEN was able to decrease NO production in LPS-induced cells. LPS + GDEN 0 as LPS control was observed to have lower NO production than other treatments, including positive control, LPS + DEX. Similar result was also reported by Hickman *et al.* that M1 macrophages produced less NO compared with unpolarized macrophages (M0), despite the expression of iNOS by M1 was measured higher than M0 and M2. From here, they concluded that high-level gene expression was not always followed by high functionality or product.

LPS-induced cells treated with 40 μg/ml GDEN had the lowest NO production than other treatments, although it was not significantly different with positive control and LPS + GDEN 0. This result suggested GDEN 40 μg/ml was a favorable concentration to inhibit NO as one of the M1 products and resolve cell inflammation. Integration with the result of MTT showed the ability of NO production per cell from LPS + GDEN 0 to LPS + GDEN 40 μg/ml treatments had the similar a trend with total NO production. NO production per cell from those treatments was measured to be 0.014; 0.016; 0.012; 0.013; and 0.008 nM/cell respectively. In addition, NO production per cell was significantly different between LPS + GDEN 0 and LPS + GDEN 40 μg/ml, but not between LPS + GDEN 7.5–20 μg/ml. This result also supported GDEN 40 μg/ml was able to provide anti-inflammatory effect more effectively than other concentrations.

### Goldenberry-derived exosome-like nanoparticles effect in RAW 264.7 polarization

**Figure 8.**
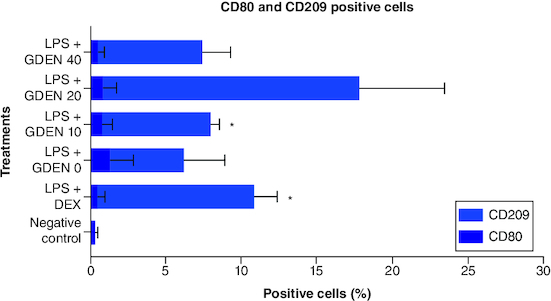
Expression of CD80 and CD209 after cells were induced by LPS and treated with various concentrations of GDEN. CD markers expression was measured by flow cytometry. Statistical results showed that there was no significant difference between treatments, except LPS + GDEN 10 and LPS + DEX toward negative control. *p-value <0.05 (n = 3).

The effect of GDEN in macrophage polarization was evaluated through the expression of CD80 as M1 marker and CD209 as M2 marker [30,31]. All data related to this study are available in Mendeley data (https://data.mendeley.com/datasets/7dkhr88t3s/1). As shown in Figure 8, treatment with GDEN showed a tendency to downregulate the expression of CD80, indicating a decrease in M1 population, although the differences were not statistically significant. Treatment with 40 μg/ml GDEN revealed a percentage of CD80 positive cells which is almost as good as positive control, LPS + DEX. Thus, it could indicate that this concentration of GDEN indeed had better potential in suppressing M1 polarization as it also synchronized with the result of NO production mentioned above.

As in the result of CD209 positive cells which represented M2 population, treatment with 20 μg/ml GDEN showed the highest result of CD209 positive cells compared with other treatments, including positive control which was treated with antiinflammation drug, dexamethasone. However, this result was measured to be not significant toward any treatments. This was probably due to the high standard deviation in the collected data. As in LPS + GDEN 10 μg/ml which had a lower mean of CD209 positive cells than LPS + GDEN 20 μg/ml, it was significant toward negative control due to the smaller standard deviation. Furthermore, CD209 positive cells from LPS + GDEN 20 μg/ml also had the smallest p-value (0.242) toward LPS + GDEN 0 (LPS control, without GDEN), while in other GDEN concentration, 10 and 40 μg/ml, the calculated p-values were 0.823 and 0.959, respectively. As the concentration of GDEN was increased to 40 μg/ml, CD209 positive cells conversely decreased. This outcome could indicate that the ability of GDEN to polarize M2 was probably affected by its concentration. Due to this limitation, it is suggested that future research should evaluate more than one marker for macrophage polarization. As M2 has been addressed to have subtypes with different phenotypes [32], assessment of more markers will provide a more thorough result when working with macrophage polarization.

**Figure 9.**
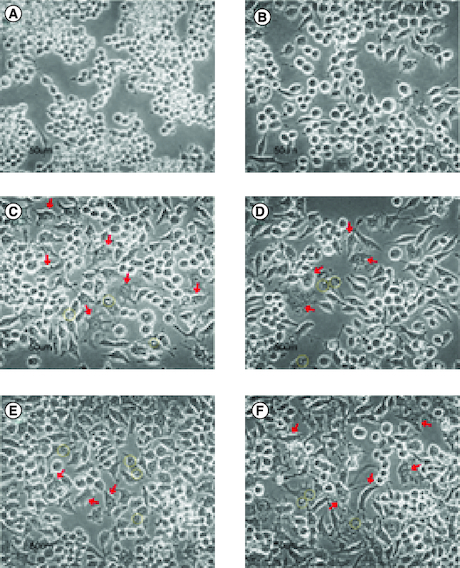
Cells morphology (400×). From negative control **(A)**, LPS + DEX **(B)**, LPS + GDEN 0 μg/ml **(C)**, LPS + GDEN 10 μg/ml **(D)**, LPS + GDEN 20 μg/ml **(E)**, and LPS + GDEN 40 μg/ml **(F).** Red arrow represented irregular-shaped cells with pseudopodia; yellow circle represented vacuolization in cell.

From microscopy observation, cells from negative control were round-shaped (Figure 9A), while LPS-induced cells tended to be long, irregular, forming pseudopodia, vacuoles, and few with more than one nucleus in a cell, represented by Figure 9C. The later morphology was observed in some cells from LPS + GDEN 10 μg/ml treatment. Irregular shape and vacuolization were also noticed in cells from LPS + GDEN 20 μg/ml, but to a lesser degree. Cells from LPS + GDEN 40 μg/ml appeared to have a longer extension of pseudopodia and more distinguishable irregular morphology than two other GDEN concentrations. On the other hand, cells from LPS + DEX treatment had less pseudopodia spreading and a rounder shape, similar to negative control. Montana & Lampiasi reported that differences in cell morphology could be related to the activation of RAW 264.7 into M1 and M2 [33]. M2 was found to be rounder than M1 which were long-shaped, both types of cells could have pseudopodia as well. These observation results could support the previous finding that LPS + GDEN 40 μg/ml had lower CD209 positive cells compared with LPS + GDEN 20 μg/ml.

## Discussion

PEG6000 12% solution, which was utilized during GDEN isolation in this study, functions to alter the solubility of extravesicular vesicles by forming mesh-like net to capture the vesicles. Hence, the vesicles will be easier to be precipitated at low centrifugation speed [24]. PEG has been used as food additive (sweetener) so it is safe to be consumed with maximum limit of 10 mg/kg body weight per day [34]. Compared with ultracentrifugation, isolation using PEG6000 provides a more economical cost and does not require specific skill qualifications to handle it. PDEN isolation using PEG6000 is reported to have no significant difference with ultracentrifugation method in the aspect of size, zeta potential, and *in vitro* bioactivity [24]. Therefore, this method is a great alternative when other sophisticated machinery is limited.

GDEN isolated in this study using the centrifuge combined with PEG6000 precipitation method had shown that its size and morphology were alike to PDEN isolated from other plants [8,11]. Isolated GDEN also were not cytotoxic to cells till concentration of 60 μg/ml and were able to be internalized by cells. These results indicate the success in GDEN isolation and its *in vitro* compatibility to RAW 264.7 cells, thus supporting its further application.

NO works as messenger molecules in nervous, cardiovascular and immune systems [1]. During inflammation, NO level will be elevated due to the activation of iNOS which is expressed by M1 macrophage [29]. Excessive production of NO can damage surrounding tissues as NO reacts with other radical molecules which are cytotoxic and induce prolonged inflammation [35]. Therefore, reduction of NO is used as anti-inflammatory indicator of a substance. NO production assay revealed that high GDEN concentration, which is 40 μg/ml, was able to suppress NO production on LPS-induced cells, indicating the anti-inflammatory effect of GDEN. The ability to decrease NO production had been reported by other studies using goldenberry extract. The ability seems to be inseparable from goldenberry's metabolites. For example, withanolides compound found in goldenberry was reported to decrease NO production by BV-2 microglia cells by targeting the residues of iNOS active site [36]. Other compounds, such as flavonoid and phenolic were identified in large amounts from goldenberry leaf extract and were able to inhibit NO production and iNOS expression on RAW 264.7 [37].

Based on CD80 and CD209 expression results, GDEN displayed the tendency to decrease M1 polarization and increase M2 population, particularly when treated with concentration of 20 μg/ml. Macrophages show plasticity in their polarization state based on the environmental stimuli they received [2]. Increase in M2 population signals inflammation resolution and promotes tissue repair since this type of macrophage produces anti-inflammatory cytokines and transforming growth factors [2,3]. Thus, enhancement of M2 polarization by 20 μg/ml GDEN supports its anti-inflammatory potential. The effect of PDEN on macrophage polarization also has been reported in turmeric, *Pueraria lobata*, and ginseng [^15-17^]. This ability of PDEN can be related to its lipid integrity and protein as reported in another study, removing protein from ginseng PDEN by proteinase and disruption of its lipid by sonication lead to the loss of its macrophage polarization ability [15]. This can be due to the important roles of protein and lipid in the uptake process [11,14]. Metabolites carried by PDEN also play a role in exhibiting its bioactivities. In goldenberry, metabolites, such as withanolides, rosmarinic acid, and physalin, have been found to have anti-inflammatory and immunomodulatory effects [36,38,39]. Extract of another species from Physalis genus is reported to contain withanolides and sucrose ester that are able to downregulate M1 marker and enhance M2 marker [40]. Physalin D is a member of Physalin group and frequently found in Physalis genus. It is reported to promote polarization of M1 into M2 and prevent M2 repolarization into M1 when treated with LPS/IFN-γ [41].

## Conclusion

Based on the results above, GDEN had been successfully isolated from goldenberry fruits and showed anti-inflammatory potential by decreasing M1 product and M1 population, along with promoting M2 polarization when treated with 20 μg/ml GDEN. The anti-inflammatory potential of GDEN is likely to be related to protein, lipid composition and metabolites in it. These findings support further study regarding GDEN and its application as anti-inflammatory drug. Additional marker evaluation for macrophage polarization is recommended for future study to ensure thorough validation.
